# Altered Posterior Cerebellar Lobule Connectivity With Perigenual Anterior Cingulate Cortex in Women With Primary Dysmenorrhea

**DOI:** 10.3389/fneur.2021.645616

**Published:** 2021-06-22

**Authors:** Xiaoyan Wu, Wenjun Yu, Xuwei Tian, Zhiying Liang, Yun Su, Zhihui Wang, Xiumei Li, Li Yang, Jun Shen

**Affiliations:** ^1^Department of Radiology, Sun Yat-Sen Memorial Hospital, Sun Yat-Sen University, Guangzhou, China; ^2^School of Psychology, South China Normal University, Guangzhou, China; ^3^Precise Genome Engineering Center, School of Life Sciences, Guangzhou University, Guangzhou, China; ^4^School of Education, Jinggangshan University, Jiangxi, China; ^5^Department of Radiology, First People's Hospital of Kashgar, Xinjiang, China

**Keywords:** primary dysmenorrhea, anterior cingulate cortex, cerebellum, functional connectivity, gray matter volume

## Abstract

**Objectives:** This study aimed to investigate the potential connectivity mechanism between the cerebellum and anterior cingulate cortex (ACC) and the cerebellar structure in primary dysmenorrhea (PDM).

**Methods:** We applied the spatially unbiased infratentorial template (SUIT) of the cerebellum to obtain anatomical details of cerebellar lobules, upon which the functional connectivity (FC) between the cerebellar lobules and ACC subregions was analyzed and the gray matter (GM) volume of cerebellar lobules was measured by using voxel-based morphometry (VBM) in 35 PDM females and 38 age-matched healthy females. The potential relationship between the altered FC or GM volume and clinical information was also evaluated in PDM females.

**Results:** PDM females showed higher connectivity between the left perigenual ACC (pACC) and lobule vermis_VI, between the left pACC and left lobule IX, and between right pACC and right cerebellar lobule VIIb than did the healthy controls. Compared with healthy controls, no altered GM volume was found in PDM females. No significant correlation was found between altered cerebellum–ACC FC and the clinical variables in the PDM females.

**Conclusion:** PDM females have abnormal posterior cerebellar connectivity with pACC but no abnormal structural changes. ACC–cerebellar circuit disturbances might be involved in the PDM females.

## Introduction

Primary dysmenorrhea (PDM) is a common gynecological disease in women of reproductive age, which is characterized by severe chronic pelvic pain and menstrual cramps but with normal pelvic anatomy (1). Sometimes, PDM can be accompanied by low back pain and sleeplessness and even result in emotional and cognitive dysfunction in the affected individuals (2). To date, its neurological basis has been reported to be predominantly related to the central nervous system (CNS) comprising the periaqueductal gray (PAG), anterior cingulate cortex (ACC), dorsomedial prefrontal cortex (PFC) (dmPFC), precuneus, insula, and amygdala, which are associated with pain perception, transmission, and modulation (3–6). For example, PDM females showed altered cortical thickness and gray matter (GM) volume in the brain region involved in the generation of negative affect and top-down pain modulation as revealed by structural magnetic resonance imaging (MRI) (5, 7–10). In addition, abnormal brain metabolism, spontaneous activity, and functional network in the default mode network (DMN) and emotional network (3–6, 11–14), which contribute to higher-level sensory and attention processing toward pain and affect regulation, have also been found in PDM females.

The ACC, a hub of the pain-related neural circuit, receives inputs from the thalamus, amygdala, and insular cortex involved in nociceptive information transfer and emotional mediation of the pain state and projects to the PFC and insular cortex involved in sensory modulation and pain-related anxiety (15). It is known that PDM can cause an increase in GM volume (8, 9) and brain activity of the ACC (4) and abnormal functional connectivity (FC) of the ACC with the other cerebral cortex (3, 4, 11, 13). The FC of the ACC subregions was also found to be abnormal, including increased connectivity between the causal ACC (cACC) and somatosensory cortex and between the subgenual ACC (sACC) and medial PFC (mPFC) in PDM females; and the disturbance of connectivity loops in different ACC subregions may reflect different function deficits such as the dysfunction of pain sensory pathway and affective pain process (11). Furthermore, the connectivity of the dorsal ACC (dACC) with the ventromedial PFC (vmPFC) (13) and connectivity of the perigenual ACC (pACC) with the precuneus/vmPFC (4, 11, 13) were reported to be involved in cognitive and emotional modulation of pain in PDM females. Although these findings underline the importance of the ACC and ACC–cerebral cortex circuits in PDM, they mainly focused on the ACC subregion connectivity with the cerebral cortex, not the cerebellum.

The cerebellum is not only recognized as a crucial region for sensorimotor control (16, 17) but also involved in high-level functions, such as pain processing, cognitive control, and emotional monitoring (18–21). Previous studies on chronic pain have already shown abnormal cerebellar function and structure associated with pain-related emotion modulation in patients with low back pain (22, 23), complex regional pain syndrome (24), and medication-overuse headache (25). The cerebellar connectivity with the ACC was also related to cognitive and emotion processing (26–29). A recent study of the rat model of recurrent headache (30) further demonstrated increased connectivity between the cerebellum and ACC, suggesting the participation of the cerebellum–ACC connectivity in the pain-related process. In PDM women, decreasing spontaneous activity in the right cerebellum posterior lobe (31) and increasing GM volume in the cerebellar tonsil (right VIIIa) (8) have been found. However, whether the cerebellum–ACC connection is aberrant and different cerebellum–ACC circuits correspond to different pain-related processes in PDM remain unknown. The role of the cerebellum in pain processing in PDM females also needs to be further elucidated.

In this study, the FC between the ACC subregions and cerebellar lobules and the GM volume of the cerebellum were analyzed in the PDM females. The relationship between altered FC and GM volume and clinical information were also examined. The purpose of this study was to determine whether the cerebellum–ACC subregion connectivity and GM volume of the cerebellum might be another neurological basis in PDM.

## Materials and Methods

### Participants

Thirty-eight right-handed women with PDM and 42 healthy women were recruited from the university by recruitment advertisement. PDM was diagnosed in Sun Yat-Sen Memorial Hospital, according to the American College of Obstetricians & Gynecologists. Inclusion criteria for PDM were as follows: (1) a regular menstrual cycle between 27 and 32 days; (2) menstrual pain is >4 on the visual analog scale (VAS; 0 = no pain, 10 = worst pain) in the recent 6 months; and (3) pelvis MRI scan did not show any anatomical pelvic disease. The inclusion criteria for the healthy controls were similar to those for the PDM females except that the VAS score is 0 in the controls. The exclusion criteria for all participants were anatomical pelvic diseases, history of neurological or psychiatric disorders, alcohol or drug dependency, pregnant, or any contraindication for MRI scan. Self-rating Depression Scale (SDS) (32) and the Self-rating Anxiety Scale (SAS) (33) were also used to evaluate emotion state in PDM women. MRI scan of all subjects was performed during the periovulatory phase (days 12–16 of the menstrual cycle), which is the phase in which influence of PDM was usually evaluated (3, 11, 34). All participants wrote informed consent, and this study was performed according to Declaration of Helsinki and approved by the ethics committee of Sun Yat-Sen Memorial Hospital, Sun Yat-Sen University.

### MR Data Acquisition

MRI was performed on a 3.0-T unit (Achieva; Philips Healthcare, the Netherland) with an 8-channel head coil in Sun Yat-Sen Memorial Hospital, Sun Yat-Sen University. Earplugs were placed in the ears to reduce scanner noise, and a foam block was used to fix the head during the scan. T2^*^-weighted fast field echo–echo-planar imaging (FFE-EPI) was performed to collect resting-state functional MRI (rs-fMRI) data. There were 240 volumes obtained in 8 min with the following acquisition parameters: repetition time (TR) = 2,000 ms, echo time (TE) = 30 ms, flip angle (FA) = 90°, field of view (FoV) = 240 × 240 mm^2^, acquisition matrix = 64 × 64, slice number = 33, and thickness = 4.0 mm without gap between slices. T1-weighted 3D FFE sequence was acquired to obtain high-resolution structural brain images with the following acquisition parameters: TR = 8.2 ms, TE = 3.7 ms, FA = 8°, FoV = 256 × 256, acquisition matrix = 256 × 256, thickness = 1 mm, and slice number = 168.

### fMRI Data Preprocessing

All rs-fMRI data were preprocessed using SPM 12 (https://www.fil.ion.ucl.ac.uk/spm/software/spm12/) and DPABI v3.1 toolbox (35) in MATLAB R2013a. First, the following steps were conducted for rs-fMRI data of each subject: removal of the first 10 functional volumes; slice timing correction; head motion correction; segmentation of the high-resolution structural images; nuisance covariate removal; normalization of whole-brain functional images to Montreal Neurological Institute (MNI) standard space; spatial smoothing [a 4-mm full width at half maximum (FWHM)] and band-pass filtering (0.01–0.1 Hz). Afterward, the quality control was performed according to the large head motion criterion (translation >2 mm in any plane, or rotation >2° in any direction) and the normalized map. Then, the mean frame-wise displacement Jenkinson index (Mean FD Jenkinson) (36) was calculated to match the head motion between the PDM females and healthy controls (*p* = 0.435). Three PDM and four healthy women with excessive head motion or bad normalization were excluded.

### Cerebellum Normalization Using the Spatially Unbiased Infratentorial Template

The cerebellum normalization was performed using the spatially unbiased infratentorial template (SUIT) toolbox (http://www.diedrichsenlab.org/imaging/suit.htm) (37, 38), which is intended to achieve more accurate cerebellum inter-subject alignment than the whole-brain methods. The SUIT is a cerebellum-specific template, including the cerebellum and brainstem structures, which can provide higher degree anatomical details of the cerebellum than the whole-brain MNI template. The use of SUIT template can improve the superposition of fissures, the spatial variance, and the overlap of the deep cerebellar nuclei and further promote the alignment of anatomical and functional areas (37–39). First, the anatomical image was transferred into LPI orientation, and the origin of the anatomical image was set to the anterior commissure. Then, the cerebellum and brainstem were isolated from the whole brain and segmented into GM and white matter. The isolation map was further checked and corrected to ensure perfect isolation using the MRIcron (https://people.cas.sc.edu/rorden/mricron/index.html), and any GM outside the cerebellum was excluded. The segmented GM images were normalized to the SUIT template using a non-linear deformation. The functional images with slice timing, head motion correction, and nuisance covariate removal were resliced according to the above-determined deformation. Finally, smoothing (4-mm FWHM) and filtering (0.01–0.1 Hz) were performed for the above functional images.

### Functional Connectivity Analysis

**Figure 1:**
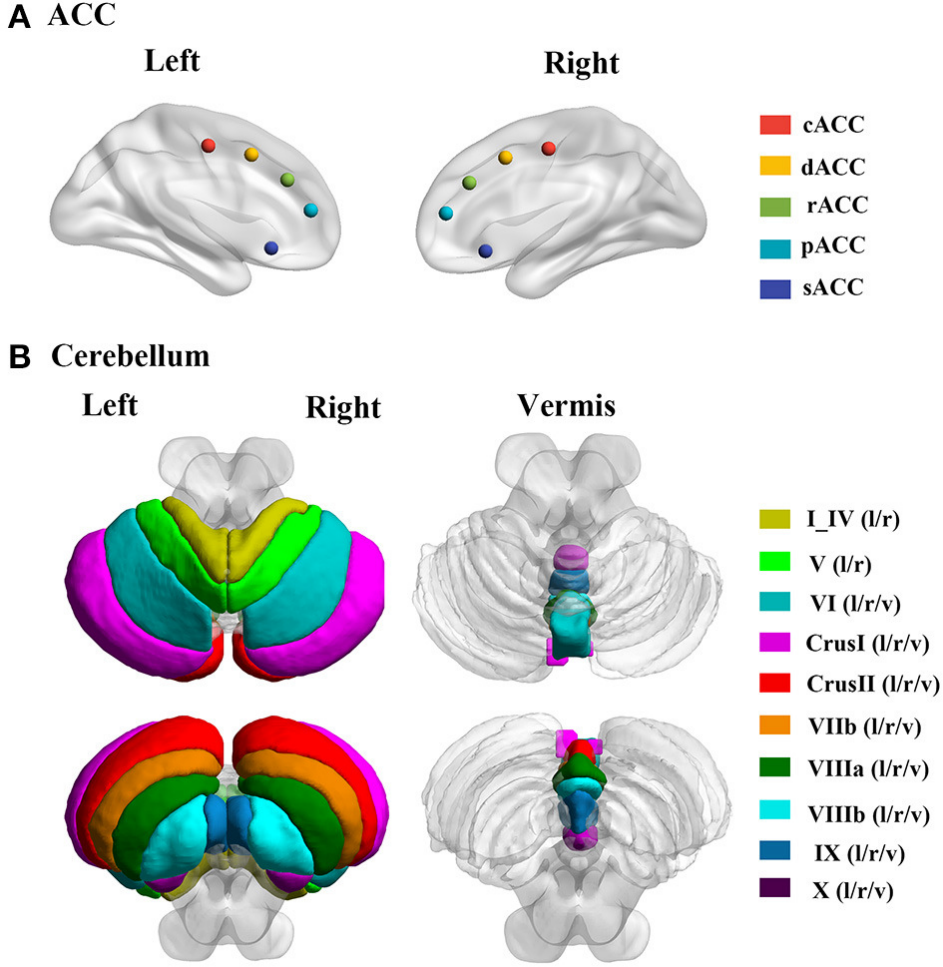
The locations of ACC and cerebellum subregions. **(A)** Five ACC subregions, including cACC, dACC, rACC, pACC, and sACC. **(B)** Cerebellum including 28 lobules; the I_IV and V including the bilateral regions; and the VI, CrusI, CrusII, VIIb, VIIIa, VIIIb, IX, and X including bilateral and vermis regions. cACC, causal anterior cingulate cortex; dACC, dorsal anterior cingulate cortex; rACC, rostral anterior cingulate cortex; pACC, perigenual anterior cingulate cortex; sACC, subgenual anterior cingulate cortex; l, left hemisphere; r, right hemisphere; v, vermis.

Region of interest (ROI)-based FC was performed for each subject. For the ACC, five spherical ROIs with 5-mm radius were placed on the most typical ACC subregions, including the cACC, dACC, rostral ACC (rACC), pACC, and sACC (40–42), in a previous PDM study (11). These ACC subregions are involved in different domain functions, such as motor control (cACC), cognitive control (dACC and pACC), conflict monitory (rACC), self-referential and social processing (pACC), and emotional regulation (sACC, pACC, and dACC) (11, 41, 43–45). These subregions were centered in the following coordinates: bilateral cACC (±5, −10, 47), dACC (±5, 14, 42), rACC (±5, 34, 28), pACC (±5, 47, 11), and sACC (±5, 25, −10). For the cerebellum, ROIs were selected according to the SUIT template, including 28 lobules (anterior lobe: bilateral I–IV and V; posterior lobe: bilateral and vermis VI, Crus I, Crus II, VIIb, VIIIa, VIIIb, and IX; flocculonodular lobe: bilateral and vermis X) (Figure 1). The average time courses of each ACC subregion and cerebellar lobule were extracted by averaging across all voxels within each ROI. The connectivity between the ACC and cerebellum was estimated by computing the correlation coefficient, and a 5 × 28 matrix was obtained for each subject. The correlation matrices were converted to z-scores by using Fisher's *r*-to-*z* transformation.

### Voxel-Based Morphometry Analysis

The GM volume of all cerebellum lobules was analyzed by using voxel-based morphometry (VBM) procedure in SPM12 platform with SUIT toolbox. In brief, after the isolation, the structural images were normalized and resliced to the SUIT template. Then, the smoothing with a 4-mm FWHM Gaussian kernel was performed to reslice structural images. Finally, the average signals of cerebellar lobules in each subject were extracted as GM volume for between-group comparison.

### Statistical Analysis

Two-sample *t*-test was used to test the between-group differences in age, menstrual beginning age, menstrual duration year, menstrual cycle, SAS, SDS, and FD Jenkinson. A non-parametric permutation *t*-test was used to test the group difference in cerebellum FC with the ACC, and cerebellar GM volume between PDM and healthy controls, with menstrual cycle as a regressor. The statistically significant threshold was set as *p* < 0.05 with a false discovery rate (FDR) correction. Furthermore, the mean value of the altered FC of cerebellum–ACC or cerebellar GM volume was extracted for each PDM. Pearson correlation analysis was used to determine the relationship between the altered cerebellum–ACC FC, cerebellar GM volume, and clinical variables, including VAS, pain duration years, SAS, and SDS.

## Results

### Demographic and Clinical Information

The demographic and clinical information of 35 PDM women (20.49 ± 1.20 years old) and 38 healthy women without PDM (20.58 ± 1.52 years old) is shown in Table 1. There were no significant differences in the age, menstrual beginning age, menstrual duration year, SAS, and SDS between PDM and healthy control groups (*p* > 0.05). The PDM group had a significantly longer menstrual cycle than the healthy control group (*p* = 0.019).

**Table 1:** Demographic and clinical information in 35 women with primary dysmenorrhea (PDM) and 38 healthy controls.

	**PDM (*n =* 35)**	**Controls (*n =* 38)**	***p*-value**
Age, years	20.49 ± 1.20	20.58 ± 1.52	0.773
Begin age of menstrual, years	12.60 ± 1.31	12.74 ± 2.60	0.780
Duration years of menstruating	7.89 ± 1.75	7.63 ± 3.04	0.667
Days of Menstrual cycle	30.46 ± 3.21	28.74 ± 2.89	0.019
Pain begin age, years	15.14 ± 1.99	N/A	N/A
Pain duration year, years	5.14 ± 2.13	N/A	N/A
Pain degree	6.54 ± 1.09	N/A	N/A
SAS	33.78 ± 5.35	31.65 ± 4.68	0.074
SDS	37.62 ± 6.71	36.77 ± 6.12	0.573

*Values represent mean ± standard deviation*.

*N/A, non-applicable; SAS, Self-Rating Anxiety Scale; SDS, Self-Rating Depression Scale*.

### Cerebellum–Anterior Cingulate Cortex Functional Connectivity

**Figure 2:**
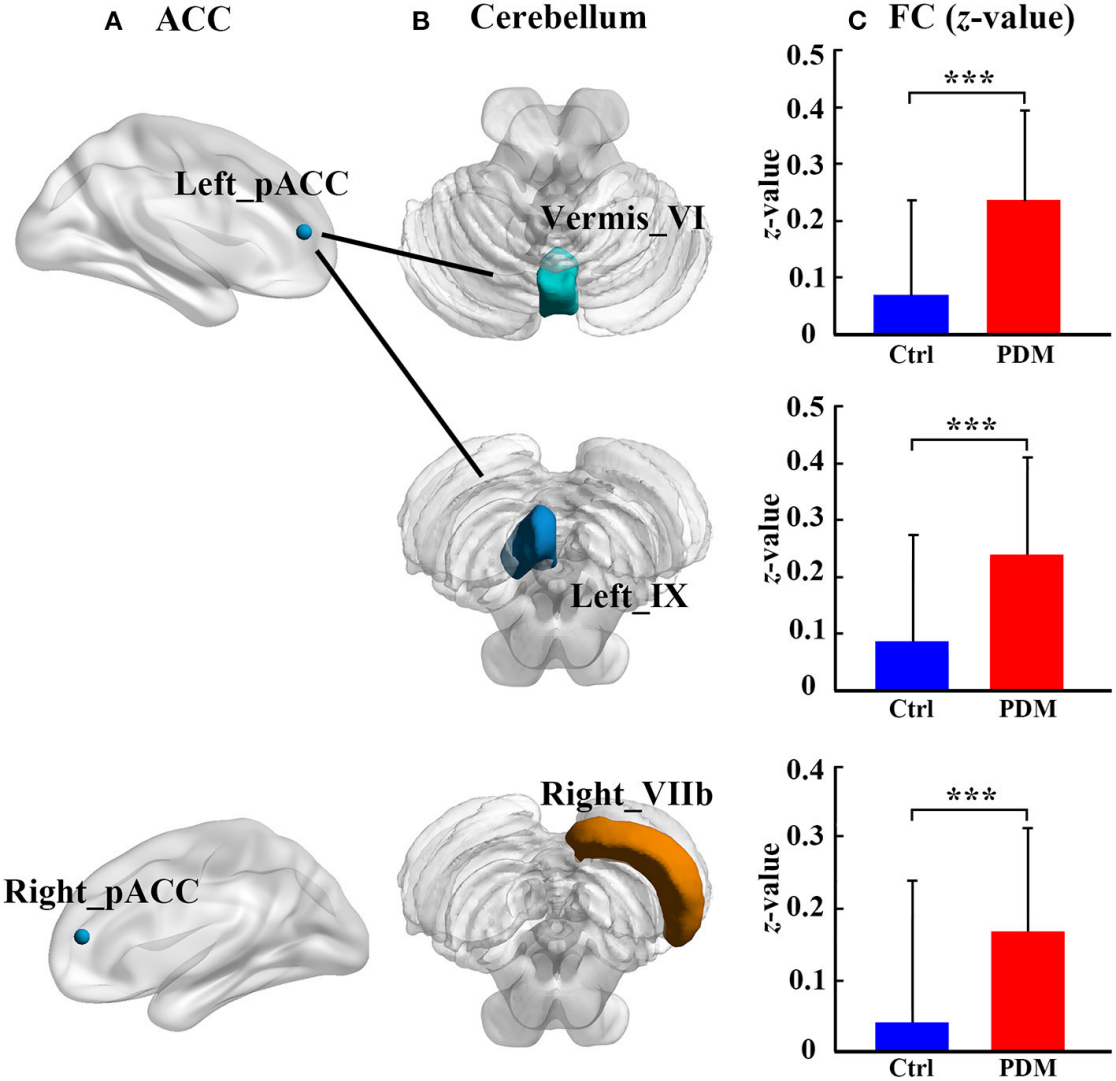
The significant difference of ACC–cerebellum FC between the PDM females and healthy controls. There are three abnormal ACC–cerebellum FC: **(A)** the ACC subregions of abnormal FC; **(B)** the cerebellar lobules of abnormal FC; **(C)** bar plot showing the FC (*z*-value) in each abnormal FC for each group, the bar height represents the mean value, and the error bar corresponds to standard deviation. FC, functional connectivity; Ctrl, healthy control; PDM, primary dysmenorrhea; pACC, perigenual anterior cingulate cortex; ***p < 0.001.

Cerebellum–ACC FC in PDM and healthy control groups is shown in Table 2. Compared with healthy controls, the PDM group had higher FC between the left pACC and cerebellar vermis_VI, between the left pACC and left cerebellar lobule IX, and between the right pACC and right cerebellar lobule VIIb (FDR correction, *p* < 0.05, Figure 2).

**Table 2:** The significant difference of ACC–cerebellum FC between PDM and healthy control.

**ACC**	**Cerebellum**	**PDM**	**Controls**	***p*-value**
Left_pACC	Vermis_VI	0.236 ± 0.160	0.069 ± 0.168	<0.001
Left_pACC	Left_IX	0.237 ± 0.169	0.086 ± 0.186	<0.001
Right_pACC	Right_VIIb	0.169 ± 0.143	0.042 ± 0.198	<0.001

*Values represent mean ± standard deviation*.

*pACC, perigenual anterior cingulate cortex; FC, functional connectivity; PDM, primary dysmenorrhea*.

### Cerebellar Lobules Gray Matter Volume

GM volumes of all cerebellar lobules in PDM and healthy control groups are shown in Table 3. We found that there was no significant difference in the GM volume between PDM females and healthy control (FDR correction, *p* < 0.05, Table 3).

**Table 3:** The significant difference of cerebellar GM volume between PDM and healthy control.

	**ROI**	**PDM**	**Controls**	***p*-value**
1	Left_I_IV	0.507 ± 0.047	0.492 ± 0.046	0.140
2	Right_I_IV	0.495 ± 0.045	0.486 ± 0.045	0.255
3	Left_V	0.592 ± 0.049	0.571 ± 0.051	0.055
4	Right_V	0.570 ± 0.048	0.552 ± 0.052	0.101
5	Left_VI	0.643 ± 0.055	0.617 ± 0.053	0.037
6	Vermis_VI	0.509 ± 0.057	0.488 ± 0.051	0.042
7	Right_VI	0.614 ± 0.058	0.589 ± 0.053	0.044
8	Left_CrusI	0.580 ± 0.061	0.560 ± 0.054	0.102
9	Vermis_CrusI	0.309 ± 0.053	0.296 ± 0.046	0.135
10	Right_CrusI	0.572 ± 0.061	0.550 ± 0.052	0.067
11	Left_CrusII	0.556 ± 0.059	0.539 ± 0.057	0.127
12	Vermis_CrusII	0.535 ± 0.052	0.525 ± 0.061	0.240
13	Right_CrusII	0.546 ± 0.064	0.536 ± 0.055	0.291
14	Left_VIIb	0.596 ± 0.060	0.575 ± 0.062	0.108
15	Vermis_VIIb	0.653 ± 0.073	0.636 ± 0.063	0.129
16	Right_VIIb	0.585 ± 0.074	0.568 ± 0.058	0.192
17	Left_VIIIa	0.595 ± 0.064	0.566 ± 0.069	0.054
18	Vermis_VIIIa	0.615 ± 0.057	0.596 ± 0.057	0.083
19	Right_VIIIa	0.584 ± 0.086	0.558 ± 0.070	0.125
20	Left_VIIIb	0.538 ± 0.063	0.515 ± 0.080	0.098
21	Vermis_VIIIb	0.630 ± 0.060	0.609 ± 0.067	0.069
22	Right_VIIIb	0.524 ± 0.079	0.505 ± 0.080	0.178
23	Left_IX	0.535 ± 0.067	0.501 ± 0.080	0.029
24	Vermis_ IX	0.657 ± 0.065	0.631 ± 0.078	0.053
25	Right_ IX	0.545 ± 0.070	0.524 ± 0.080	0.118
26	Left_I	0.431 ± 0.054	0.422 ± 0.057	0.345
27	Vermis_ I	0.409 ± 0.043	0.392 ± 0.045	0.037
28	Right_ I	0.375 ± 0.055	0.381 ± 0.054	0.261

*Values represent mean ± standard deviation*.

*GM, gray matter; PDM, primary dysmenorrhea; ROI, region of interest*.

### Correlation Analysis

The correlations between the altered cerebellum–ACC FC and the clinical variables in the PDM group are shown in Table 4. Correlation analysis showed no significant correlations between the altered cerebellum–ACC FC and clinical information in the PDM females (*p* > 0.05, Table 4).

**Table 4:** Correlation between cerebellum–ACC FC and clinical variables in the PDM females.

	**Pain duration**		**Pain severity (VAS)**		**SAS**		**SDS**	
	***r***	***p***	***r***	***p***	***r***	***p***	***r***	***p***
Left_pACC –Vermis_VI	0.095	0.588	0.196	0.260	0.111	0.524	0.096	0.582
Left_pACC –Left_IX	8.63e−04	0.996	−0.070	0.688	0.209	0.229	0.095	0.589
Right_pACC –Right_VIIb	0.148	0.395	0.260	0.131	0.111	0.526	−0.034	0.845

*ACC, anterior cingulate cortex; pACC, perigenual ACC; VAS, visual analog scale; SAS, Self-rating Anxiety Scale; SDS, Self-rating Depression Scale*.

## Discussion

Our study results showed that PDM females had higher connectivity between the left pACC and vermis_VI, between the left pACC and left lobule IX, and between the right pACC and right lobule VIIb, than had healthy controls. Altered cerebellum–ACC FCs were not correlated with clinical variables in the PDM females. There were no significant group differences in the GM volume. These aberrant circuits between the ACC subregions and specific cerebellar lobules extend the knowledge of the cerebellum function in pain processing, and cognitive and emotional function of PDM, also intensify the multidimensionality of pain processing. A cerebellum-specific template was used in our study to normalize and segment cerebellar structure. To the best of our knowledge, this is the first study to use the SUIT cerebellar template to examine the cerebellar mechanism of pain processing in PDM.

In our study, the FC between the ACC subregions and cerebellar lobules was analyzed by using SUIT method to comprehensively describe the potential aberrant ACC–cerebellar circuit. The results showed that PDM females had higher FC between the right pACC and right cerebellar lobule VIIb, between the left pACC and cerebellar vermis_VI, and between the left pACC and left cerebellar lobule IX. Previously, the pACC not only participated in self-referential and social processing (43) but also is involved in cognitive processing, the same as the dACC (45) and emotion regulation via the sACC (44–47). In addition, the pACC was also thought to be related to the pain control machine (48–51). As for PDM, Liu et al. (4, 11) has found that abnormal pACC connectivity with precuneus and caudate was associated with pain duration and severity in the PDM women. In our study, the abnormal pACC connectivity with cerebellar lobules was found to be mainly located in the posterior cerebellum, including lobule VIIb, vermis_VI, and lobule IX. These regions are related to high-level functions, such as cognitive function, memory processing, emotion monitoring (17, 19, 52, 53). For example, cerebellar lobule VIIb is seen as a “cognitive cerebellum,” which contributes to the specific cerebro-cerebellar loops that participate in the executive control task (54–57). Both cerebellar vermis and lobule IX are seen as “limbic cerebellum,” which is functionally connected with limbic brain structures that are involved in emotional processing (17, 20, 58). Moreover, the posterior cerebellum lesion (lobule VII/vermis) can deprive cerebro-cerebellar-cognitive/limbic loops of cerebellar input (52, 59). These loops are associated with attention deficit (59), affective alterations (19), and reduced emotional expressivity (60). This phenomenon has not been reported in chronic pain. Previously, individuals with chronic low back-related leg pain (22) and low back pain (23) showed changed local connectivity and GM volume in the posterior cerebellum. Furthermore, the altered spontaneous activity (31) and glycometabolism (61) in the right posterior cerebellum lobe were detected in the PDM patients that further supported the results of abnormal posterior cerebellar connectivity in our study. Taken together, abnormal connectivity between the pACC and posterior cerebellum may reveal dysfunction in the ACC–cerebellar cognitive and emotion processing circuit in PDM. However, in our study, no correlation was found between the altered cerebellum–ACC FC and clinical variables, including VAS, pain duration years, SAS, and SDS, in the PDM females. Notably, in our study, VAS, SAS, and SDS were assessed in the pain-free phase rather than in the menstruation phase (pain phase). This might result in an underestimation of VAS, SAS, and SDS.

In our study, the cerebellar GM volume was further calculated for the cerebellar structure. Compared with healthy controls, PDM females had no altered GM volume. Previously, Tu et al. (8) have reported increased GM volume of the posterior cerebellum (cerebellar tonsil) in PDM females based on the whole-brain standard MNI template. This discrepancy might be due to different template normalization and the duration of history of PDM. In the study by Tu et al., PDM females had a PDM history of 10.31 ± 3.30 years. The PDM females in our study had a history of 5.14 ± 2.13 years. A longer history of pain may lead to more severe adverse effect on cerebellar structure in the PDM females.

There was a limitation in our study. PDM women were included under the pain-free phase, not under the pain phase. Previous studies (6, 9, 12) have shown different brain functional and structural alterations in PDM women between the pain phase and pain-free phase. Whether different pain phases can exert a distinct effect on the cerebellar lobules needs to be determined in the future study.

## Conclusion

In summary, our study demonstrated aberrant cerebellum–ACC FC but unaltered cerebellar GM volume in the PDM females based on the cerebellum-specific SUIT template. PDM females could have increased connectivity between the left pACC and vermis_VI, between the left pACC and left lobule IX, and between the right pACC and right lobule VIIb. ACC–cerebellar circuit disturbances might be involved in the PDM females.

## Data Availability Statement

The raw data supporting the conclusions of this article will be made available by the authors, without undue reservation.

## Ethics Statement

The studies involving human participants were reviewed and approved by Ethics Committee of Sun Yat-Sen Memorial Hospital, Sun Yat-Sen University. The patients/participants provided their written informed consent to participate in this study.

## Author Contributions

MRI data acquisition and behavioral testing were conducted by XT, WY, ZL, YS, ZW, and XL. Data analysis were performed by XW, ZL, and YS. Literature search and the first draft of the manuscript was written by XW. JS and LY involved in supervision, guided the experiments, and revised the manuscript. All authors read, approved the final manuscript, contributed to the study conception, design, and commented on previous versions of the manuscript.

## Conflict of Interest

The authors declare that the research was conducted in the absence of any commercial or financial relationships that could be construed as a potential conflict of interest.
